# Aspiration-Related Deaths in 57 Consecutive Patients: Autopsy Study

**DOI:** 10.1371/journal.pone.0103795

**Published:** 2014-07-30

**Authors:** Xiaowen Hu, Eunhee S. Yi, Jay H. Ryu

**Affiliations:** 1 Department of Respiratory Medicine, Anhui Provincial Hospital Affiliated to Anhui Medical University, Hefei, China; 2 Division of Anatomic Pathology, Mayo Clinic, Rochester, Minnesota, United States of America; 3 Division of Pulmonary and Critical Care Medicine, Mayo Clinic, Rochester, Minnesota, United States of America; Fundación Jimenez Diaz, Spain

## Abstract

**Background:**

Aspiration can cause a diverse spectrum of pulmonary disorders some of which can lead to death but can be difficult to diagnose.

**Patients and Methods:**

The medical records and autopsy findings of 57 consecutive patients in whom aspiration was the immediate cause of death at Mayo Clinic (Rochester, MN, USA) over a 9-yr period, from January 1 2004 to December 31 2012 were analyzed.

**Results:**

The median age at death was 72 years (range, 13–95 years) and included 39 (68%) males. The most common symptom before death was dyspnea (63%) and chest radiography revealed bilateral infiltrates in the majority (81%). Most common precipitating factors for aspiration were depressed consciousness (46%) and dysphagia (44%). Aspiration-related syndromes leading to death were aspiration pneumonia in 26 (46%), aspiration pneumonitis in 25 (44%), and large airway obstruction in 6 patients (11%). Aspiration was clinically unsuspected in 19 (33%) patients. Antimicrobial therapy had been empirically administered to most patients (90%) with aspiration pneumonia and aspiration pneumonitis.

**Conclusion:**

We conclude aspiration-related deaths occur most commonly in the elderly with identifiable risks and presenting bilateral pulmonary infiltrates. One-third of these aspiration-related pulmonary syndromes were clinically unsuspected at the time of death.

## Introduction

Aspiration is defined as inhalation of oropharyngeal or gastric contents into the larynx and lower respiratory tract [1]. Aspiration can lead to a broad spectrum of pulmonary diseases such as airway obstruction, pneumonia, chemical pneumonitis or acute respiratory distress syndrome with significant morbidity and mortality [2], [3]. These syndromes are often misdiagnosed and may lead to suboptimal management [4]. Recent studies suggest aspiration is often unsuspected in patients with aspiration-related pulmonary syndromes [5], [6]. Detailed characterization of patients dying from aspiration-related pulmonary syndromes has previously not been performed but may help identify patients at risk and improve diagnosis to avoid preventable deaths. In the current study, we analyzed 57 consecutive postmortem examinations performed at a tertiary-referral medical center on patients dying from aspiration-related pulmonary syndromes to characterize their clinical and radiologic presentations and the rate of misdiagnosis.

## Methods

### Study subjects

Using a computer-assisted search of medical records we identified 57 patients undergoing an autopsy at the Mayo Clinic (Rochester, MN, USA) over a 9-year period from January 1, 2004 to December 31, 2012 and were identified to have aspiration-related pulmonary disease as the immediate cause of death on postmortem examination. This cohort comprised 1.2% of 4,623 autopsies that included examination of the lungs over this period, Aspiration-related pulmonary syndromes encountered in this cohort included aspiration pneumonia, aspiration pneumonitis and large airway obstruction. Aspiration pneumonia was diagnosed by: 1) clinical and radiologic features of pneumonia (cough, dyspnea, fever, and evolving radiologic infiltrates) and 2) pathologic findings of aspiration (presence of foreign material) and acute bronchopneumonia sometimes accompanied by microabscesses and granulomatous inflammation at postmortem examination. Aspiration pneumonitis was diagnosed by: 1) acute onset of respiratory symptoms with new pulmonary infiltrates on chest imaging and 2) pathologic evidence of acute lung injury (diffuse alveolar damage and hemorrhagic pulmonary edema) combined with the presence of foreign material or foreign body giant cell reaction at postmortem examination. Large airway obstruction was defined as acute respiratory failure related to the presence of large particulate material causing large airway obstruction confirmed on postmortem examination. Patients who died from drowning and amniotic fluid aspiration were excluded. Other forms of aspiration-related pulmonary syndromes including diffuse aspiration bronchiolitis and exogenous lipoid pneumonia were not encountered in this autopsy study. We identified the time of aspiration event based on available medical history as well assessment of clinical course and emergence of new pulmonary infiltrates on chest imaging. The current study was approved by the Mayo Foundation Institutional Review Board (Rochester, MN, USA).

### Clinical Data

Medical records were examined in detail and the following data were retrieved: age, sex, clinical presentation, risks for aspiration, radiologic findings, events leading up to death, and autopsy findings. We also determined clinician's diagnosis as to the cause of death based on review of all available medical records including clinical notes as well as the clinician diagnoses listed on death certificates. The clinician diagnosis and the type of aspiration-related pulmonary syndrome causing death was assigned based on a review of all available data by consensus of the two authors (XH and JHR).

### Statistical Analyses

Continuous data are presented as median and range (minimum, maximum), when appropriate, and frequency, percentages for categorical variables.

## Results

Of 57 patients with aspiration-related deaths, 39 (68%) were male (**Table 1**). The median age was 72 years (range, 13 to 95 years). Twenty seven of these patients (47%) were hospitalized at the time of aspiration; the remainder included 8 (14%) whose aspiration likely occurred in the nursing home or other institutional facility and 22 (39%) at home.

**Table 1:** Demographic and Clinical characteristic of 57 patients with aspiration-related deaths.

Characteristic		Value
**Sex, n (%)**		
	Male	39 (68)
	Female	18 (32)
**Age, yrs**		
	Median	72
	Range	13–95
**Main symptoms, n (%)**		
	Dyspnea	36 (63)
	Decreased consciousness	11 (19)
	Hypotension	4 (7)
	Cardiorespiratory arrest	2 (4)
	Cough	1 (2)
	Febrile	1 (2)
	No symptom	2 (4)

One or more precipitating factors for aspiration were identified in all 57 patients and mainly included depressed consciousness in 26 patients (46%), dysphagia (as determined by clinical observation or formal swallow study) in 25 patients (44%); 6 patients had two risk factors (**Table 2**). Specifically, sedation accounted for 13 (50%) of those with depressed consciousness and neurological disorders were present in 21 (84%) of those with dysphagia including dementia (7 subjects) and Parkinson's disease (3 subjects). In their review on aspiration pneumonia in the elderly, Marik and Kaplan [7] identified dysphagia and impaired cough reflex associated with neurologic diseases as the dominant factors increasing the likelihood of aspiration in this population.

**Table 2:** Precipitating factors in 57 aspiration related deaths.

Risk factor	N (%)
Depressed consciousness	26 (46)
Dysphagia	25 (44)
Vomiting	5 (9)
GERD	4 (7)
Compromised airway defense*	3 (5)

*Compromised airway defense consisted of vocal cord immobility, oropharyngeal deformities caused by surgery or radiation therapy, and endotracheal intubation.

The type of aspiration-related pulmonary syndrome leading to death were classified as aspiration pneumonia in 26 (46%), aspiration pneumonitis in 25 (44%), and large airway obstruction in 6 (11%). All six large airway obstruction cases died within 72 hours of aspiration. Among the 19 aspiration pneumonitis cases in whom the time of aspiration event could be identified retrospectively, 14 (74%) died within 72 hours. In contrast, among 22 cases of aspiration pneumonia in whom the time of initial aspiration event could be identified, only 6 (27%) died within 72 hours.

Among 27 patients who suffered aspiration in the hospital were 10 aspiration pneumonia, 14 aspiration pneumonitis and 3 large airway obstruction cases. In the remaining 30 patients with aspiration likely occurring outside of the hospital were 16 aspiration pneumonia, 11 aspiration pneumonitis and 3 large airway obstruction cases.

Main symptoms recorded preceding death included dyspnea (63%), decreased consciousness (19%) (**Table 1**). Sudden onset of severe illness related to aspiration occurred in seventeen patients (30%); 10 patients had aspiration pneumonitis, 6 patients had large airway obstruction, and one remaining patient had aspiration pneumonia.

Fifty-three patients (93%) underwent chest radiography, 16 of whom also had undergone chest computed tomography (CT); four patients had no chest imaging available prior to death. Bilateral distribution of parenchymal abnormalities was seen in 44 patients (83%). The predominant radiologic findings were patchy alveolar infiltrates and/or atelectasis in 46 patients (87%); remaining seven patients exhibited mixed patterns of interstitial, consolidative, and ground-glass opacities. These chest imaging findings were relatively nonspecific.

During the hospital course, 35 patients underwent invasive mechanical ventilation (non-invasive ventilation in two patients including one with aspiration pneumonia and another with aspiration pneumonitis); 20 patients had aspiration pneumonitis, 14 patients had aspiration pneumonia and, 1 patient had large airway obstruction. Extracorporeal membrane oxygenation was also employed in one patient with aspiration pneumonitis. Bronchoscopy was performed in 16 patients, including one procedure performed at an outside hospital and one patient who underwent the procedure twice. Three of these patients were found to have evidence of aspiration and 12 patients underwent the procedure for clearance of airway secretion; the bronchoscopic inspection was normal in the remaining patient.

Microbial culture results were available in 19 patients (15 respiratory secretions, one lung biopsy specimen, and 3 autopsy specimens) with aspiration pneumonia, nine of whom had potential pathogens identified including *Staphylococcus aureus* (four patients including two patients who also had *Enterobacter species and Pseudomonas aeruginosa, respectively*), *Enterobacter* species (two patients), *Streptococcus*(one patient), *Nocardia* (one patient), *Klebsiella pneumoniae complex* coexisting with *Enterococcus* (one patient), and *Candida* (one patient with lymphoma). Microbial culture results were available in 14 patients with aspiration pneumonitis (11 respiratory secretions, and four autopsy specimens), 9 of whom had potential pathogens identified including *Staphylococcus aureus* (three patients, one coexisting with *Escherichia coli* and *Enterobacter* species, one coexisting with *Pseudomonas aeruginosa*), *Escherichia coli* (three patients, one coexisting with *Klebsiella* species), *Enterobacter* species (two patients, one coexisting with *Haemophilus* species), *Klebsiella* species (two patients), *Citrobacter koseri* (one patient) and *Streptococcus* species (one patient). Antimicrobial therapy had been administered to 23 patients (88%) with aspiration pneumonia and 22 patients (92%) with aspiration pneumonitis, including three in each group treated with antifungal drugs.

Aspiration was included among diagnostic possibilities by clinicians at the time of death for 38 patients (67%). Nineteen patients (33%) whose aspiration was clinically unsuspected included nine who died from aspiration pneumonitis, nine with aspiration pneumonia, and one with large airway obstruction (**Table 3**). Clinician's diagnoses for immediate cause of death for these patients most commonly included pneumonia (five patients), neurologic disorders (four patients), sepsis (three patients), and heart failure (two patients).

**Table 3:** Undiagnosed Aspiration Cases (n = 19): Comparison of Postmortem and Clinician Diagnoses.

Postmortem diagnosis	Clinician diagnosis	N
Aspiration pneumonitis (n = 9)		
	Pneumonia	3
	Sepsis	2
	Acute subdural hematoma	1
	Esophageal cancer	1
	Heart failure	1
	Neutropenic fever	1
Aspiration pneumonia (n = 9)		
	Pneumonia	2
	Alzheimer's dementia	1
	Esophageal rupture	1
	Fall with concussion	1
	Heart failure	1
	Respiratory failure with ALS	1
	Sepsis	1
	Virus encephalitis	1
Large airway obstruction (n = 1)		
	Cardiac arrest	1
**Total**		**19**

ALS = amyotrophic lateral sclerosis.

## Discussion

With the exception of few reported cases, aspiration-related deaths have not been methodically investigated [8], [9]. Our study is the largest autopsy analysis examining aspiration-related deaths and demonstrates aspiration to be clinically unsuspected as the cause of death in one-third of such cases. Aspiration pneumonia and aspiration pneumonitis were encountered in nearly equal portions and together accounted for 90% of these deaths. Large airway obstruction caused the remainder of aspiration-related deaths.

Our data also show that dysphagia related to neurologic disorders comprise the commonest precipitating factor for aspiration-related deaths, followed by depressed consciousness related to sedation. These findings are in agreement with prior studies showing neurologic disorders and sedation to be common precipitating factors for aspiration [3], [4], [10]. Wada et al [11] reported aspiration pneumonia in patients with Alzheimer's disease to be independently associated with severity of dementia. In patients with stroke, choking is estimated to have resulted in 36,997 deaths in the United States between 2001 and 2010 [9]. Although aspiration associated with general anesthesia has been strikingly reduced over the years [12], use of sedative medications contributed to 23% of deaths in our study.

Dyspnea was the most common symptom recorded for patients in this study and is similar to a prior study describing the presence of this symptom in almost half of 36 aspiration cases confirmed on lung biopsy specimens [13]. Bilateral lung involvement in chest CT was found in more than half of 53 hospitalized patients with aspiration pneumonia in a recent study [14]. Our results suggest a higher frequency of bilateral lung involvement based on radiography and autopsy findings compared to the prior study. The difference may be explained by more severe lung disease in our cohort.

Aside from large airway obstruction-related death which is very familiar to clinicians [15], [16], there are two common entities of aspiration pulmonary syndromes, aspiration pneumonia and aspiration pneumonitis. Mylotte and colleagues estimated 40% suspected pneumonias in nursing home residents to be aspiration pneumonitis based on definite or suspected aspiration event [17], [18]. Their results are similar to our data showing aspiration pneumonitis to account for 44% of aspiration-related deaths. Aspiration pneumonitis may be difficult to diagnose in the absence of witnessed aspiration since its presentation and clinical course can be similar to progressive bacterial pneumonia.

According to our data, aspiration pneumonia is the most common type of aspiration-related pulmonary syndrome leading to death. There were more patients with underlying neurological disorders in our study for whom the median age was 72 years old compared to two prior studies [19], [20]. Chang et al. reported aspiration pneumonia as a cause of death in 5% of patients with stroke [9]. Both neurological disorder and old age have been recognized as risk factors for aspiration pneumonia [4], [19].

Our data demonstrated the common isolated pathogens in aspiration pneumonia were *Staphylococcus aureus*, *Enterobacter* species rather than anaerobes. This was similar to that previously reported in the literature [18], [19], [21], [22]. On the other hand, a recent study showed the predominant pathogenic bacteria of aspiration pneumonia in patients admitted to a medical intensive care unit to be antibiotic-resistant bacteria [21]. However, it should be noted that standard respiratory cultures obtained at autopsy did not include anaerobic cultures.

Pathogenic bacteria were recovered in a portion of patients with aspiration pneumonitis, a disease entity generally thought to involve aspiration of sterile gastric contents. There are several potential explanations for this finding. Any form of aspiration is likely to include entrance of oral secretions into the lung. Emerging data on human microbiota have demonstrated that multiple species of bacteria can exist in the stomach as well as lungs of healthy individuals [23], [24]. Furthermore, tube feeding and acid-suppressive pharmacologic therapies promote bacterial growth [4].

Irwin and colleagues [25] reported eight of 14 (57%) sudden deaths due to food asphyxiation to have been misdiagnosed as acute myocardial infarction. In another study, aspiration was suspected clinically in only 9% of 59 patients in whom aspiration was confirmed on lung biopsy for evaluation of undiagnosed lung disease [13]. In our study, one third of aspiration related deaths were unsuspected before autopsy. Co-existing medical conditions in the elderly population and relatively nonspecific presentations of aspiration-related syndromes likely contributed to misdiagnosis.

Aside from the issue of correctly diagnosing aspiration-related syndromes, our study has some implications on the prevention of aspiration-related deaths. The patients at risk appear to be identifiable such as those with neurologic impairment and dysphagia. Optimal strategies in managing patients identified to be at risk generally require multidisciplinary expertise in maintaining adequate hydration and nutrition while minimizing the risks of aspiration. These options many include tube feeding, oral and bronchial hygiene measures, positioning, antimicrobial and other pharmacologic therapies [2], [3], [4]. Heightened awareness is needed in considering aspiration as the cause of an undiagnosed pulmonary process in patients at risk with prompt and appropriate investigative maneuvers to achieve earlier diagnosis and appropriate management.

This study included consecutive patients undergoing autopsy during a nine year period and the diagnostic criteria were based on combined clinical and autopsy findings. We, however, acknowledge that the limitations of this study including the retrospective design which limited the analysis to clinical data available in medical records and autopsy results. There are likely many patients who died from aspiration but did not undergo a postmortem examination and were undiagnosed. There is a selection bias in autopsy studies which tend to enrich for atypical and diagnostically challenging cases. Microbiology data were limited to standard respiratory cultures obtained at postmortem examination and may not have recovered some pathogens such as anaerobic bacteria. Furthermore, aspiration pneumonia and aspiration pneumonitis can sometimes be difficult to distinguish.

## Conclusions

Aspiration-related deaths most commonly occur in the elderly with identifiable risks and often manifesting bilateral pulmonary infiltrates. In one-third of these patients aspiration as the cause of death was unsuspected by clinicians.
